# Poly(ADP-ribosyl)ation is involved in the epigenetic control of *TET1* gene transcription

**DOI:** 10.18632/oncotarget.1905

**Published:** 2014-04-17

**Authors:** Fabio Ciccarone, Elisabetta Valentini, Maria Giulia Bacalini, Michele Zampieri, Roberta Calabrese, Tiziana Guastafierro, Germano Mariano, Anna Reale, Claudio Franceschi, Paola Caiafa

**Affiliations:** ^1^ Department of Cellular Biotechnologies and Hematology, “Sapienza” University of Rome, Rome, Italy; ^2^ Pasteur Institute-Fondazione Cenci Bolognetti, Rome, Italy; ^3^ Department of Experimental Pathology, Alma Mater Studiorum, University of Bologna, Bologna, Italy

**Keywords:** poly(ADP-ribosyl)ation, TET1, DNA methylation, 5-hydroxymethylcytosine

## Abstract

TET enzymes are the epigenetic factors involved in the formation of the sixth DNA base 5-hydroxymethylcytosine, whose deregulation has been associated with tumorigenesis. In particular, TET1 acts as tumor suppressor preventing cell proliferation and tumor metastasis and it has frequently been found down-regulated in cancer. Thus, considering the importance of a tight control of *TET1* expression, the epigenetic mechanisms involved in the transcriptional regulation of *TET1* gene are here investigated. The involvement of poly(ADP-ribosyl)ation in the control of DNA and histone methylation on *TET1* gene was examined. PARP activity is able to positively regulate *TET1* expressionmaintaining a permissive chromatin state characterized by DNA hypomethylation of *TET1* CpG island as well as high levels of H3K4 trimethylation. These epigenetic modifications were affected by PAR depletion causing *TET1* down-regulation and in turn reduced recruitment of TET1 protein on *HOXA9* target gene. In conclusion, this work shows that PARP activity is a transcriptional regulator of *TET1* gene through the control of epigenetic events and it suggests that deregulation of these mechanisms could account for *TET1* repression in cancer.

## INTRODUCTION

Ten-Eleven translocation (TET) family enzymes, namely TET1, TET2 and TET3, are new important epigenetic regulators of gene expression [1]. Their epigenetic action depends on the ability to convert 5-methylcytosine (5mC) primarily to 5-hydroxymethylcyosine (5hmC) and then to 5-formylcytosine or 5-carboxylcytosine [2]. These sequential modifications of 5mC are initial steps in active DNA demethylation processes based on DNA repair pathways [1,3]. However, 5hmC is actually considered a stable epigenetic mark of DNA other than an intermediate of active DNA demethylation [1,4,5]. In fact, TET proteins modulate DNA methylation and transcription acting in embryonic development, stem cell function and differentiation [4,6]. TET1 and TET2 participate in the DNA demethylation during germline development [7] while the epigenetic reprogramming of mammalian zygote mainly depends on TET3 enzymatic activity [8]. Furthermore, TET1 and TET2 are involved in stem cell biology promoting transcription of pluripotency factors and repressing developmental regulators [9-11]. Besides producing 5hmC and its derivatives, TET enzymes are involved in epigenetic events also controlling O-linked β-N-acetylglucosaminylation (GlcNAcylation) and in turn histone H3K4 methylation [12,13].

The importance of TET enzymes is also demonstrated by the pathological consequences deriving from their deregulation [6]. Dysfunction of TET enzymatic activity can be accompanied by a decrease in 5hmC levels which is now considered an epigenetic hallmark of human cancers [14-16]. TET1 and TET2 generally act as tumor suppressors regulating cancer development, growth and invasion [17-19]. Accordingly, frequent somatic mutations in *TET2* gene have been identified in hematopoietic malignancies [20,21]. Rare genetic anomalies were also identified for *TET1* gene [22-24] but down-regulation of *TET1* has more frequently been observed during transformation [14,18,25]. However, the epigenetic mechanisms necessary for a tight control of *TET1* expression are largely unknown and thus they are here investigated.

Attention has been focused on poly(ADP-ribosyl)ation (PARylation), a reversible post-translational modification catalysed by PARP family enzymes which covalently introduce ADP-ribose polymer (PAR) chains onto acceptor proteins [26,27]. Notably, target proteins can also undergo non-covalent modification by PARs when bringing specific PAR-binding modules [28-30]. Another important feature of PARylation is the automodification reaction according to which some PARP enzymes, primarily the founding member PARP-1 (also known as ARTD1) [31] and its highly homologous protein PARP-2/ARTD2, synthesize PARs on themselves [26,30,32]. PARP-1 has historically been studied for its functions in DNA damage response but it plays important roles in transcriptional regulation driving epigenetic events [30,33,34]. In fact, PARP-1 enzyme orchestrates chromatin dynamics acting on histones and DNA methylation machinery [33,35,36]. PARP-1 loosens chromatin structure modifying core and linker histones [33,36] but it also modulates the catalytic activity of chromatin enzymes as observed for the covalent modification of the histone demethylases KDM5B and KDM4D [37,38]. Also non-covalent interaction with PARs can regulate enzymatic activities as demonstrated for the inhibition of the DNA methyltransferase Dnmt1 [39] or the activation of the nucleosome-remodeling ATPase ALC1 [40,41]. On the basis of this evidence, this paper investigated the involvement of PARylation in the control of *TET1* highlighting a key contribution of PARP activity in the regulation of both DNA and histone methylation.

## RESULTS

### DNA methylation and histone H3K4 trimethylation control TET1 expression

**Figure 1:**
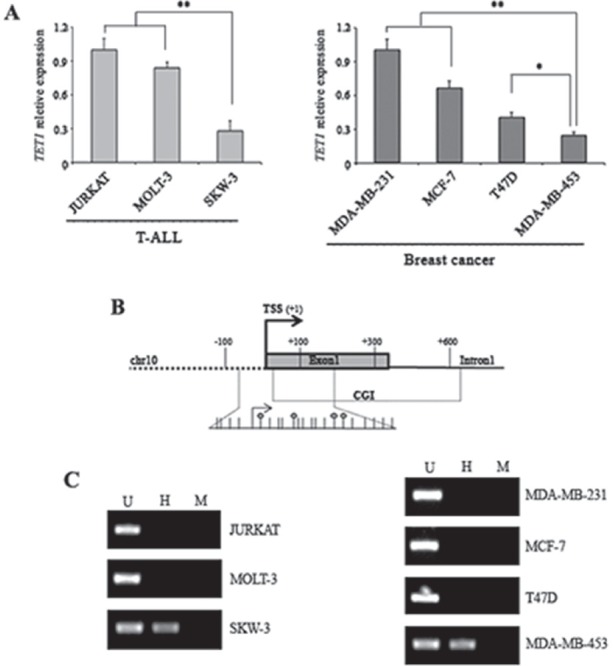
DNA hypermethylation of *TET1* CpG island associates with low *TET1* expression (A) qRT-PCR analysis of *TET1* gene expression in T-ALL and breast cancer cell lines. The results are shown as means ± S.E.M. (n=3). P-value was determined by One-way ANOVA test followed by Tukey post test (*P<0.05; **P<0.01). (B) Schematic representation of *TET1* gene where TSS and CGI are indicated. The expanded region corresponds to the fragment amplified by PCR after endonuclease restriction for DNA methylation analysis. Each stick is a CpG and each circle represents the internal CpG in the 5′-CCGG-3′ tetranucleotide recognized by both MspI and HpaII. (C) Analysis of *TET1* DNA methylation using MspI/HpaII restriction and PCR amplification performed on T-ALL and breast cancer cell lines. U, uncut; H, HpaII; M, MspI.

Expression analysis of *TET1* gene was performed on human T-cell acute lymphoblastic leukemia (T-ALL) cell lines (JURKAT, MOLT-3, SKW-3) and on cell lines of human breast cancer origin (MDA-MB-231, MCF7, T47D, MDA-MB-453). *TET1* expression was detectable in all samples tested but transcription levels were quite different. SKW-3 and MDA-MB-453 showed the lowest *TET1* expression out of T-ALL and breast cancer cell lines, respectively (Figure 1A). Considering the presence of a CpG island (CGI) located close to the transcription start site (TSS) of *TET1* gene (Figure 1B), the methylation state of *TET1* CGI in each cell line was investigated. DNA was digested with the methylation-sensitive endonuclease HpaII followed by PCR amplification of a CGI region containing HpaII restriction sites. Amplification product was only obtained for *TET1* low expressing cell lines (SKW-3 and MDA-MB-453) thus indicating that their CGI was methylated (Figure 1C).

**Figure 2:**
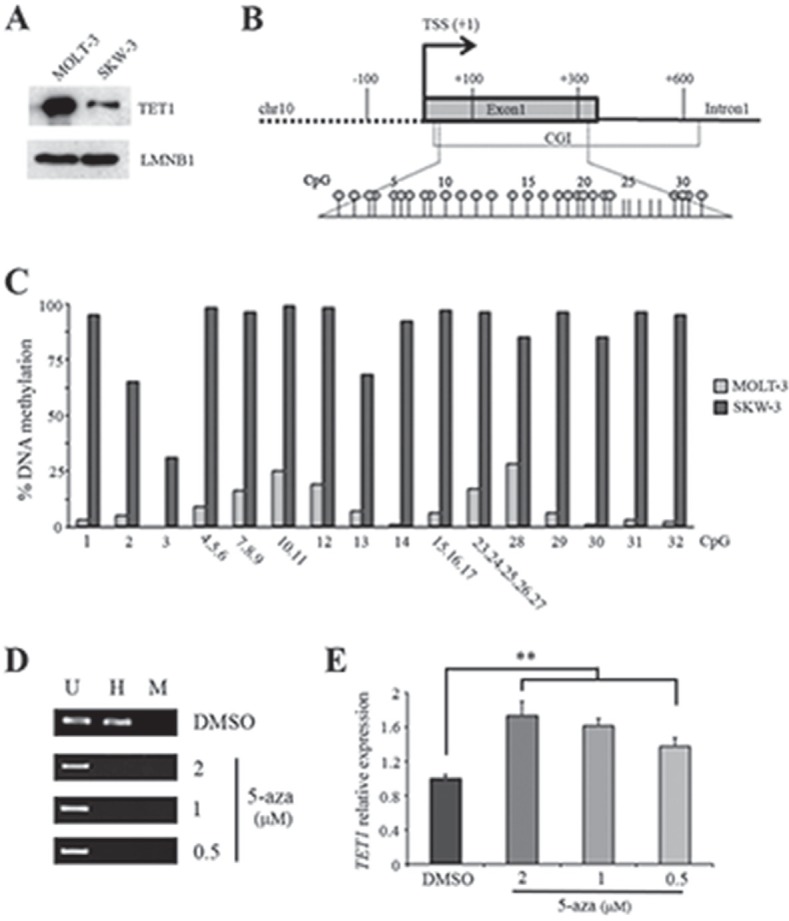
DNA methylation negatively regulates *TET1* transcription (A) Western blot analysis of TET1 protein in MOLT-3 and SKW-3 cell lines. LAMIN B1 (LMNB1) was used as loading control. (B) Schematic representation of *TET1* gene where the expanded region is the fragment investigated by EpiTYPER assay for DNA methylation analysis. Each stick is a CpG and each circle represents CpGs detectable by EpiTYPER assay. (C) DNA methylation analysis of *TET1* CGI performed by using EpiTYPER assay. (D) Analysis of *TET1* DNA methylation using MspI/HpaII restriction and PCR amplification performed on SKW-3 cells treated for 48 hrs with different doses of 5-azacytidine (5-aza). U, uncut; H, HpaII; M, MspI. (E) qRT-PCR analysis of *TET1* gene expression on SKW-3 cells treated for 48 hrs with different doses of 5-aza. The results are shown as means ± S.E.M. (n=3). P-value was determined by paired Student's t-test (**P<0.01).

To gain insight into *TET1* gene regulation, analysis was focused on two cell lines differing in the transcriptional activity of *TET1* gene: MOLT-3 cells (high *TET1* expression) and SKW-3 cells (low *TET1* expression). Accordingly, western blot analysis demonstrated that TET1 protein level in these cell lines was consistent with mRNA expression (Figure 2A). Epigenetic characterization of *TET1* promoter in MOLT-3 and SKW-3 cells was focused on DNA and histone methylation. MassARRAY EpiTYPER was used to assess DNA methylation profile of *TET1* gene demonstrating that low *TET1* expression in SKW-3 was associated with heavily methylated *TET1* CGI (Figure 2B and C). However, EpiTYPER does not discriminate between 5mC and 5hmC [42], thus experiments with glucosyltransferase enzyme were performed [43]. The absence of PCR amplification following glucosyltransferase reaction and MspI restriction demonstrated that *TET1* CGI in SKW-3 cells was methylated and not hydroxymethylated (Supplementary Figure S1A). On the basis of these results, the effective involvement of DNA methylation in the repression of *TET1* was demonstrated by treating SKW-3 cell line with the demethylating agent 5-azacytidine (5-aza). In fact, after 48 hrs of treatment, demethylation of *TET1* CGI was observed together with a dose-dependent increase in *TET1* expression (Figure 2D and E; Supplementary Figure S1B).

As concerns histone methylation, the presence of typical active (H3K4 trimethylation) and inactive (H3K9 trimethylation) chromatin marks was examined on *TET1* promoter. ChIP experiments demonstrated that H3K4 trimethylation was highly enriched in MOLT-3 with respect to SKW-3 (Supplementary Figure S1C) while H3K9 trimethylation was undetectable in both cell lines (data not shown). These results are in agreement with the transcriptional state of *TET1* in MOLT-3 and SKW-3.

### PARylation is involved in the transcriptional control of *TET1* gene

The potential involvement of PARylation in the regulation of *TET1* gene was examined as PARP activity was shown to influence *TET1* expression in germline [44] and it is able to control DNA and histone methylation [35,37,38,45,46].

**Figure 3:**
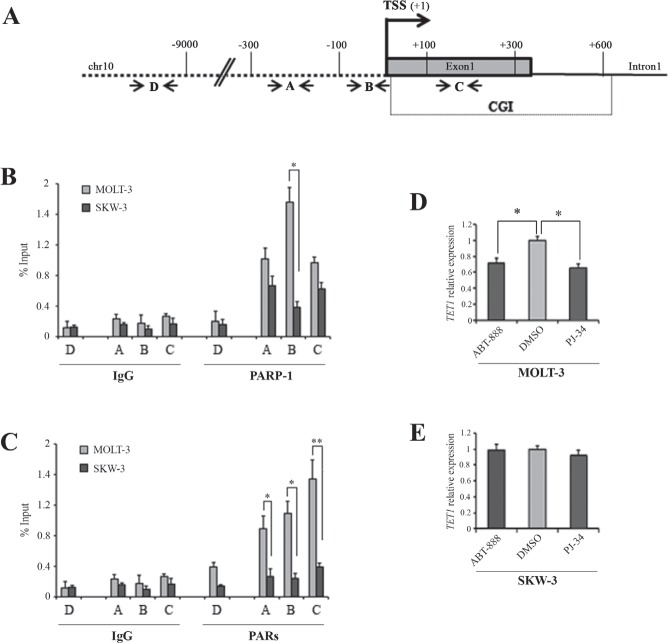
PARylation is involved in the transcriptional regulation of *TET1* gene (A) Schematic representation of *TET1* gene where arrows indicate the localization of fragments analyzed by ChIP assay. Fragment A is localized in promoter region, fragment B is very close to TSS, fragment C is in CGI region. Fragment D is used as negative control. (B,C) Analysis of PARP-1 and PARs presence on *TET1* gene regulative regions performed by ChIP assay followed by qPCR in MOLT-3 and SKW-3 cell lines. The results are shown as percentage of input and are means ± S.E.M. (n=3). (D,E) qRT-PCR analysis of *TET1* gene expression performed after treatment of MOLT-3 and SKW-3 cell lines with 1μM ABT-888 and 1μM PJ-34 for 72 hrs. The results are shown as means ± S.E.M. (n=4). P-value was determined by paired Student's t-test (*P<0.05; **P<0.01).

ChIP analysis was used to investigate the presence of PARP-1 and its enzymatic products, PARs, on regulative regions of *TET1* gene as promoter (fragment A), TSS (fragment B) and CGI (fragment C). A distal region was used as negative control (fragment D) (Figure 3A). This analysis revealed that PARP-1 and PARs were highly enriched in unmethylated *TET1* gene in MOLT-3 cells with respect to methylated *TET1* gene in SKW-3 (Figure 3B and C). Analysis of PARP-1 expression, PAR levels and DNA methylation profile of *PARP-1* CGI indicated that the different localization of PARP-1/PAR on *TET1* gene in the two cell lines was not due to general differences in PARP-1 expression or activity (Supplementary Figure S2). ChIP assay was also performed on additional cell lines: HEK293T, which showed high *TET1* expression/demethylated CGI, and HeLa showing low *TET1* expression/methylated CGI (Supplementary Figure S3A, B and C). Also in this case PARP-1 protein and PARs were mainly present on the *TET1* gene with high expression and unmethylated CGI (Supplementary Figure S3D and E).

The effective involvement of PARylation in *TET1* transcription was assessed using two potent PARP inhibitors, PJ-34 and ABT-888, at concentrations not affecting cell growth (Supplementary Figure S4). Transcriptional analysis showed that *TET1* gene expression was significantly decreased in MOLT-3 cell after treatment with 1μM PJ-34 and 1μM ABT-888 indicating the involvement of PARP activity in the positive regulation of unmethylated *TET1* gene (Figure 3D). Notably, no effect on *TET1* transcription was instead observed when PARP inhibition was performed in SKW-3 cells (Figure 3E) which showed low PARP-1/PARs content on *TET1* gene and methylated *TET1* CGI. Confirmatory findings were obtained in the highly transfectable HEK293T cell line following overexpression of the PAR-degrading enzyme PARG which led to down-regulation of *TET1* gene (Supplementary Figure S5A and B). Moreover, the silencing of PARP-1 and/or PARP-2 also reduced *TET1* transcription (Supplementary Figure S5C and D).

### PARylation controls methylation of both DNA and histone H3K4 on *TET1* gene

**Figure 4:**
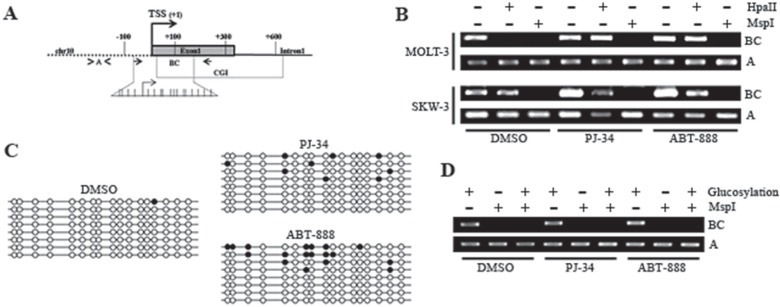
PARylation regulates DNA methylation patterns of *TET1* CpG island (A) Schematic representation of *TET1* gene where fragments BC (arrows) and A (arrowheads), amplified by PCR after endonuclease restriction for DNA methylation analysis, are indicated. Fragment BC contains recognition sites for MspI/HpaII which are not present in fragment A. The expanded region corresponds to the fragment analyzed by bisulfite sequencing where each stick is a CpG dinucleotide. (B) Analysis of *TET1* DNA methylation using MspI/HpaII restriction and PCR amplification performed on MOLT-3 and SKW-3 treated for 72 hrs with 1μM PJ-34 or 1μM ABT-888. Fragment A was used as loading control. (C) Analysis of *TET1* DNA methylation by bisulfite sequencing performed on MOLT-3 treated for 72 hrs with 1μM PJ-34 or 1μM ABT-888. Each clone is represented by a row, and the CpG dinucleotide being investigated is arranged in columns. White and black circles represent unmethylated and methylated/modified cytosines, respectively. (D) Analysis of DNA hydroxymethylation of *TET1* CGI performed using glucosyltransferase reaction and MspI restriction followed by PCR amplification. Fragment A was used as loading control. Analysis was performed on MOLT-3 treated for 72 hrs with 1μM PJ-34 or 1μM ABT-888.

The way of action of PARylation on *TET1* expression was investigated focusing on epigenetic events driven by PARP activity [33]. Attention was initially addressed to DNA methylation. Analysis of MOLT-3 cells revealed that *TET1* CGI became insensitive to HpaII cleavage after treatment with both PJ-34 and ABT-888 (Figure 4B and Supplementary Figure S6A). No changes were instead observed in SKW-3 cells whose *TET1* CGI was already methylated (Figure 4B). The addition of new methyl groups on *TET1* CGI of MOLT-3 cells in absence of PARs was then evidenced by bisulfite sequencing (Figure 4C). Glucosyltransferase-based experiments finally demonstrated that 5mC and not 5hmC was introduced onto *TET1* CGI after PARP inhibition (Figure 4D).

**Figure 5:**
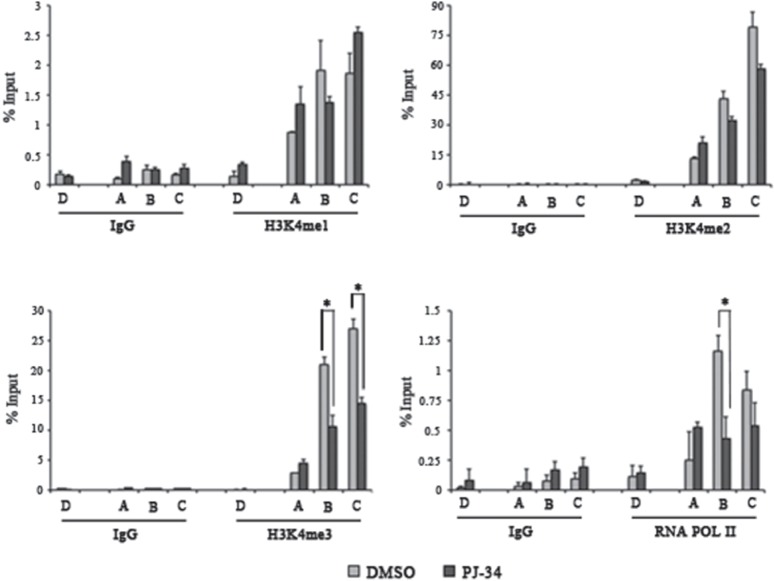
PARylation controls histone H3K4 trimethylation and RNA POL II recruitment on gene ChIP assay followed by qPCR performed on *TET1* regulative regions in MOLT-3 cells treated for 72 hrs with 1μM PJ-34. Localization of analyzed fragments is shown in figure 3A. Antibodies against mono-, di-, trimethyl-histone H3K4 and RNA POL II were used. The results are shown as percentage of input and are means ± S.E.M. (n=3). P-value was determined by paired Student's t-test (*P<0.05).

Besides DNA methylation, PARP activity is able to control histone H3K4 and H3K9 trimethylation [37,38]. Considering the absence of H3K9 trimethylation in both MOLT-3 and SKW-3, the effect of PAR depletion was assessed on H3K4 methylation states. MOLT-3 cells were treated with PJ-34 and the efficacy of PARP inhibition was demonstrated by ChIP assay with anti-PAR antibody (Supplementary Figure S6B). PAR removal was associated with a consistent decrease in H3K4 trimethylation occupancy, while no particular change was observed in H3K4 mono- and dimethylation (Figure 5). The recruitment of the RNA polymerase II (RNA POL II) was also investigated demonstrating less loading of this enzyme at the TSS of *TET1* gene in PARP-inhibited MOLT-3 cells (Figure 5). Notably, PJ-34 had no effect regarding H3K4 methylation states on *TET1* gene in SKW-3 cells (Supplementary Figure S7).

### PARylation regulates TET1 protein level and recruitment on *HOXA9* target gene

**Figure 6:**
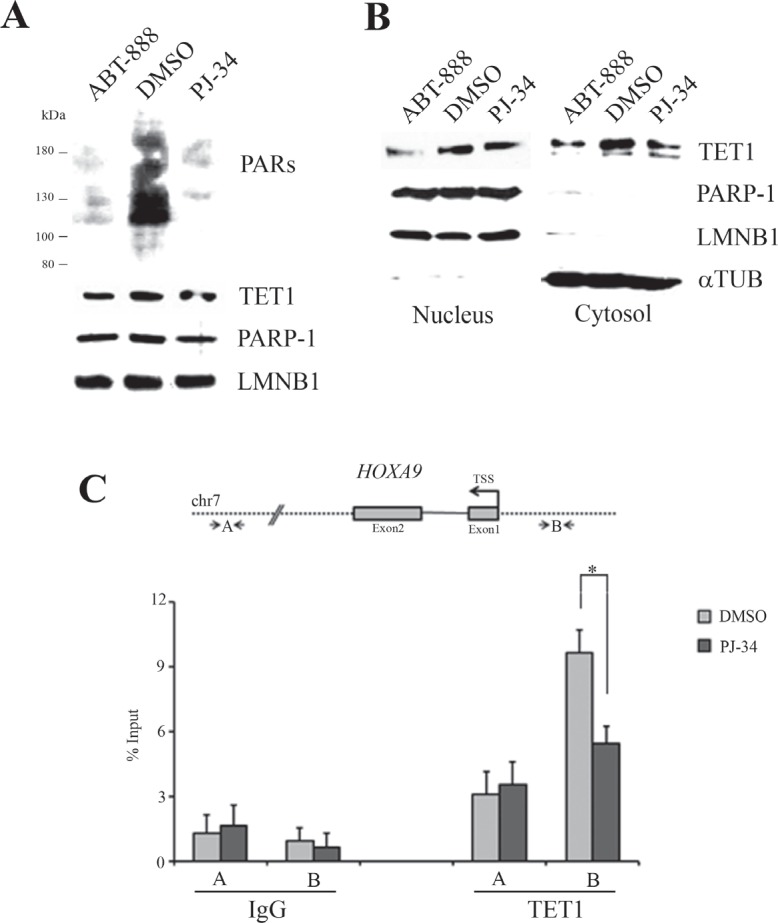
TET1 protein level and its recruitment on *HOXA9* target gene are affected by PARP inhibition (A) Western blot analysis performed on total protein lysates of MOLT-3 cells treated for 72 hrs with 1μM PJ-34 or 1μM ABT-888. LAMIN B1 (LMNB1) was used as loading control. (B) Western blot analysis of nuclear and cytosolic fractions obtained from MOLT-3 cells treated for 72 hrs with 1μM PJ-34 or 1μM ABT-888. LMNB1 and *α*TUBULIN (*α*TUB) were used as loading controls. (C) Schematic representation of TET1 target gene *HOXA9* where localization of primer A and B used in ChIP analysis is shown. ChIP assay followed by qPCR was performed using anti-TET1 antibody in MOLT-3 cells treated for 72 hrs with 1μM PJ-34. Fragment B is a known target region of TET1 protein while fragment A is a negative control. The results are shown as percentage of input and are means ± S.E.M. (n=3). P-value was determined by paired Student's t-test (*P<0.05).

Considering that PARP inhibition affects the transcription of *TET1* gene, TET1 protein level was assessed in MOLT-3 cells after PJ-34 and ABT-888 treatment. Western blots and related densitometric analyses of total, nuclear and cytosolic fractions showed a slight decrease in TET1 protein level after PARP inhibition consistently with mRNA down-regulation (Figure 6A, and Supplementary Figure S8). A possible effect of PARP inhibition on TET1 protein functions was assessed by ChIP assay on *HOXA9* gene, a known target of TET1 enzyme [19]. Notably, the recruitment of TET1 on *HOXA9* target region was affected after depletion of PARs (Figure 6C).

## DISCUSSION

TET1 protein acts as tumor suppressor gene regulating critical pathways involved in cell proliferation and tumor metastasis [19,47]. Despite rare genetic mutations of *TET1* gene having been identified [22-24], association between *TET1* down-regulation and 5hmC decrease has more frequently been observed in tumorigenesis [14,18,25]. The cause of *TET1* down-regulation in tumors is largely unknown, thus potential epigenetic mechanisms underlying *TET1* transcriptional deregulation in cancer cells have been here investigated.

Analysis was initially addressed to DNA methylation, a well-known epigenetic modification able to repress expression of tumor suppressor genes [48]. *TET1* expression was shown to be negatively regulated by methylation of the CGI located at the 5′ of the gene [19]. Analyses were here extended to several cancer cell lines and indicated that hypermethylation of *TET1* CGI was only able to reduce *TET1* transcription without causing complete silencing. Accordingly, no paper has reported a complete repression of *TET1* in cancer but only its down-regulation [14,18,25].

The study of *TET1* regulation continued comparing cell lines with different levels of *TET1* expression to characterize chromatin environment associated with *TET1* transcriptional state. MOLT-3 and SKW-3 were selected as model cell lines with high and low *TET1* expression, respectively. High-throughput quantitative DNA methylation analysis performed by MassARRAY EpiTYPER showed that *TET1* CGI was heavily methylated in SKW-3 cells with respect to MOLT-3. However, EpiTYPER is a bisulfite-based technology and thus it does not discriminate between 5mC and 5hmC [42]. Therefore, *locus*-specific analysis of DNA hydroxymethylation was performed demonstrating that the *TET1* CGI of SKW-3 cells was methylated and not hydroxymethylated. All in all, these results indicated that residual *TET1* expression in SKW-3 cells is not dependent on 5hmC and confirmed that DNA methylation of *TET1* CGI is able to reduce *TET1* gene expression.

In agreement with *TET1* transcriptional state, analysis of histone methylation evidenced higher enrichment of the active transcription mark H3K4 trimethylation in MOLT-3 with respect to SKW-3, while no signal for H3K9 trimethylation was detected. Therefore, the presence in SKW-3 of H3K4 trimethylation, which was demonstrated to permit active transcription of genes marked by DNA methylation [49], and the absence of the repressive chromatin mark H3K9 trimethylation finally clarified why *TET1* is still expressed despite the methylation of its CGI.

Considering that a connection between PARylation and *TET1* transcription was suggested [44], the molecular mechanism(s) underling this regulation was investigated. Attention was focused on the ability of PARylation to regulate transcription orchestrating epigenetic events. In fact, PARP activity was shown to protect CpGs from aberrant DNA methylation [50-53] and, apart from directly modifying histone tails [54], PARylation regulates chromatin structure impairing the activity of histone demethylases [37,38].

The action of PARylation was initially assessed analysing the presence of PARP-1 and PARs on regulative regions of *TET1* gene. ChIP experiments demonstrated that PARP-1 and PARs were mainly enriched in MOLT-3 where *TET1* gene is highly transcribed and its CGI is unmethylated with respect to SKW-3. These observations and the absence of differences in PARP-1 expression and activity in both cell lines indicated that the low amount of PARs on *TET1* in SKW-3 is not dependent on global dysfunction of PARylation. This suggests that possible cofactors of PARP-1 may account for a specific regulation of PARP activity on *TET1* gene [36,55,56]. More specifically, interacting partners of PARP-1 responsible for its recruitment and/or activation on *TET1* gene may be deregulated in specific tumor cell lines thus affecting PARP-1 positive regulation of *TET1* transcription.

Although standard ChIP protocol was demonstrated to induce PARP activity [57], in ChIP experiments here reported, PAR levels were mainly observed on *TET1* unmethylated CGI suggesting, however, that an effective difference in PARP-1 presence and/or PAR formation exists in cell lines with opposite *TET1* transcription level.

The importance of PARP activity in the regulation of *TET1* was demonstrated by both molecular and chemical approaches. Overexpression of PARG as well as silencing of PARP-1 and PARP-2 led to *TET1* down-regulation. Notably, the silencing of only PARP-1 or only PARP-2 had the same effect on *TET1* regulation of the combined silencing of both proteins. This result seems to suggest that PARP-1 and PARP-2 have a cooperative role in *TET1* regulation rather than a compensatory one. Chemical inhibition of PARylation was performed by treatment with two potent competitive PARP inhibitors, PJ-34 and ABT-888. *TET1* gene expression was reduced after PARP inhibition only in MOLT-3 cells while *TET1* in SKW-3 cells was not affected.

Notably, combination of several methods permitted the demonstration that *TET1* down-regulation in MOLT-3 cells after PAR depletion was associated with anomalous methyl groups onto *TET1* CGI. Such a results is likely to depend on the inhibitory role played by PARs on DNA methyltransferase activity [39] for the maintenance of CpG methylation-free states [33,35].

In addition to the introduction of some methyl groups on *TET1* CGI in MOLT-3 cells, PARP inhibitor treatment caused the specific reduction of H3K4 trimethylation and less recruitment of RNA POL II on *TET1* gene. All in all, these results, which are in agreement with the observed down-regulation of *TET1* in PAR-depleted MOLT-3, confirm the involvement of PARs in the inhibition of H3K4 demethylase activity and in the loading of mRNA transcription machinery [30,37]. Notably, the absence of changes after PARP inhibition on H3K4 methylation state in SKW-3 cells, which showed very low PAR content on hypermethylated *TET1* gene, further indicates a positive role played by PARylation in the regulation of *TET1* expression.

The effects of PARP inhibition on *TET1* transcription were associated with a reduction of TET1 protein levels. In particular, this reduction was observed in both nuclear and cytosolic compartments. Molecular consequences occurring after PARP inhibition were investigated analysing TET1 enrichment on a known target gene as the tumor suppressor *HOXA9* [19]. Notably, reduced recruitment of TET1 on *HOXA9* gene was evidenced in absence of PARylation. This result could depend on the reduced TET1 protein level even though a specific recruitment of TET1 mediated by PARs has also been suggested [58] and cannot be excluded.

**Figure 7:**
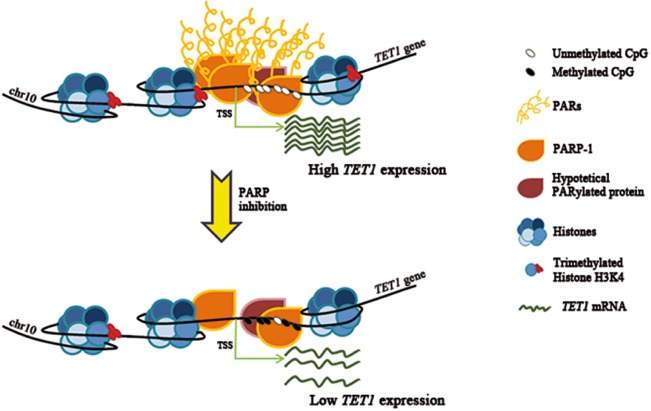
Model illustrating the way of action of PARylation in the control of *TET1* transcription PARylation of PARP-1 or other hypothetical target proteins is able to preserve the unmethylated state of *TET1* CGI as well as the presence of histone H3K4 trimethylation which are required for high *TET1* transcription. Following deregulation of PARP activity, *TET1* CGI undergoes hypermethylation and H3K4 trimethylation levels decrease causing *TET1* gene down-regulation.

In conclusion, these results highlight for the first time that PARylation can orchestrate epigenetic events on a single *locus* combining its control on both DNA and histone H3K4 methylation (Figure 7). Notably, this complex regulative network mediates a tight transcriptional control of the new epigenetic factor *TET1* in somatic cells. Therefore, PARP deregulation could be responsible for aberrant *TET1* down-regulation in cancer, as also observed for *p16(INK4a)*, *p19ARF* and *p53* tumor suppressor genes [59-62].

## Material and Methods

### Cell cultures and treatment

Human T-cell acute lymphoblastic leukemia cell lines (JURKAT, MOLT-3, SKW-3) were cultured in high glucose RPMI (Sigma-Aldrich) containing 10% FBS (Sigma-Aldrich). Breast cancer cell lines (MDA-MB-231, MCF7, T47D, MDA-MB-453), HEK293T and HeLa cells were grown in high glucose DMEM (Sigma-Aldrich) containing 10% FBS (Sigma-Aldrich). All culture solutions were supplemented with 2 mM L-glutamine (Sigma-Aldrich) and 50 U/ml Penicillin–Streptomycin (Sigma-Aldrich). Treatments of cells were performed replacing medium every 24 hrs with the indicated inhibitors PJ-34 (Sigma-Aldrich, final concentration 1 μM and 5 μM), ABT-888 (Enzo Life Sciences, final concentration 1 μM and 5 μM) and 5-azacytidine (Sigma-Aldrich, final concentrations 0.5; 1; 2 μM).

### Transfection of HEK293T cells

HEK293T cells were transfected with Lipofectamine 2000 reagent (Life Technologies) adopting the manufacturer's protocol. For PARG overexpression, empty vector PCS2 (CTRL) and pCS2-Myc-PARG (MYC–PARG) construct (51) were used. For silencing experiments, cells were transfected with siGENOME SMARTpool *PARP-1* and *PARP-2* siRNA and siGENOME Non-Targeting siRNA (Thermo Scientific, Dharmacon) at a final concentration of 50 nM.

### RNA extraction and quantitative RT-PCR

Total RNA was isolated from cells by using RNeasy mini kit (Qiagen) following the manufacturer's instructions. DNA contamination was eliminated by RNase-free DNase (Qiagen) treatment. RNA concentration and quality were evaluated by spectrophotometer analysis and agarose gel electrophoresis. Total RNA was subjected to reverse transcription using SuperScript VILO cDNA Synthesis Kit (Life Technologies). Transcriptional analysis was performed by quantitative RT-PCR (qRT-PCR) using iCycler IQ detection system (Bio-Rad). For quantitative PCR reactions, Taqman Gene Expression Assays (reported in Supplementary Methods) and EXPRESS qPCR Supermix Universal (Life Technologies) were used. Measurement of gene expression was performed using the comparative cycle threshold method. For an accurate expression analysis, geometric mean of two reference gene expressions was used for normalization. Housekeeping genes used were *β-glucuronidase* (*GUSB*) and *β-Actin* (*ACTB*).

### Antibodies

The following monoclonal antibodies were used: PARP-1 (clone C2-10; Enzo Life Sciences), PARP-2 (Enzo Life Sciences), PAR (clone 10HA; Trevigen), TET1 (Genetex), Myc (9E10 clone, hybridoma-conditioned medium), αTUB (Sigma-Aldrich). The following polyclonal antibodies were used: LMNB1 (Abcam), PARP-1 (Enzo Life Sciences), TET1 (Millipore), PAR (Trevigen), RNA POL II (Santa Cruz Biotech.), H3K4me1 (Millipore), H3K4me2 (Millipore), H3K4me3 (Millipore).

### Western blot analysis

Total cell lysates were prepared in RIPA buffer (50 mM Tris-HCl at pH 7.4, 150 mM NaCl, 1% NP-40, 0.5% sodium deoxycholate, 0.1% SDS and 1 mM EDTA) and normalized for protein concentration. Cytosolic fractions were obtained recovering supernatant after centrifugation of cells previously incubated (15 min in ice) in isolation buffer (10 mM Tris-HCl at pH 7.8, 4 mM MgCl_2_, 1 mM EDTA, 0.5 mM DTT, 1% Triton X-100, 0.25 M Sucrose). Pelleted nuclei were washed once in isolation buffer without Triton X-100, centrifuged and lysated in RIPA buffer. Each buffer was supplemented with protease inhibitor cocktail (complete EDTA-free, Roche Applied Science). Protein extracts were resolved by SDS-PAGE, transferred onto Hybond-ECL nitrocellulose membranes (Amersham Biosciences) and probed with the indicated antibodies. Densitometric analysis was performed using Quantity One software.

### ChIP assay

ChIP analysis was performed as previously described [51] with the following modifications for cells grown in suspension culture. Cells were cross-linked using 1% formaldehyde in complete medium with gentle shaking on a rotary shaker for 10 min. To quench cross-linking reaction, glycine was added to a final concentration of 0.125 M and cells were incubated on rotary shaker for 5 min. After centrifugation and two PBS washes, 1×10^6^ cross-linked cells were pelleted and then used for each immunoprecipitation condition.

### Genomic DNA isolation and analysis of DNA methylation by methylation-specific digestion combined with PCR or bisulfite modification sequencing

Genomic DNA was isolated from cells by using the DNeasy Blood and Tissue Kit (Qiagen) following the manufacturer's instructions. For detection of *TET1* CpG island methylation, genomic DNA was subjected to digestion with the methylation-sensitive enzyme HpaII (New England Bioloabs) and the methylation-insensitive enzyme MspI (New England Bioloabs) for 16 hrs at 37°C. Products from digestion were diluted and used as template for PCR reactions performed by using 5 PRIME MasterMix (5 PRIME) with specific primers (reported in Supplementary Methods). For bisulfite sequencing assay, genomic DNA was converted by using EZ DNA Methylation Kit (Zymo research). Amplification of the region of interest in *TET1* CpG island was performed using 5 PRIME MasterMix (5 PRIME) and a specific primer pair (reported in Supplementary Methods). Fragment cloning was performed using TOPO TA-cloning vector (Life Technologies) and individual inserts from randomly selected clones were sequenced.

### EpiTYPER assay for quantitative DNA methylation analysis

The EpiTYPER assay (Sequenom) was used to quantitatively analyze the DNA methylation state of *PARP-1* and *TET1* CGIs. 1 μg of DNA was bisulfite-converted using the EZ-96 DNA Methylation Kit (Zymo Research) with the following modifications: incubation in CT buffer was performed for 21 cycles of 15 min at 55°C and 30 sec at 95°C and elution of bisulfite-treated DNA was performed in 100 μl of water. PCR was performed on 10 ng of bisulfite-treated DNA using specific primers (reported in Supplementary Methods). The two *TET1* amplicons mapped in regions chr10:70,320,085-70,320,271 and chr10:70,320,251-70,320,466 respectively, while *PARP-1* amplicon mapped in chr1:226,595,508-226,596,006 region. After data cleaning, it was possible to measure the methylation state of 27 and 31 CpGs in *TET1* and *PARP-1* CGIs respectively.

### CpG-specific analysis of DNA hydroxymethylation

Site-specific analysis of 5hmC was performed through glucosylation and digestion of genomic DNA with MspI (New England Bioloabs). Briefly, DNA was treated with 5hmC glucosyltransferase (Zymo research) for 16 hrs at 30°C and then subjected to MspI restriction. After enzymatic digestion, PCR reaction was performed by using 5 PRIME MasterMix (5 PRIME) and specific primer pairs (reported in Supplementary Methods).

### Statistical Analysis

Statistical tests used and the number of replicates (n) performed are reported in figure legends. Data were considered to be statistically signiﬁcant if *P<0.05 and **P<0.01.

## SUPPLEMENTARY MATERIAL FIGURES


