# Supplementary material for: Poly(ADP-ribosyl)ation is involved in the epigenetic control of TET1 gene transcription
Source: Oncotarget. 2014 Apr 17;5(21):10356–67. doi: 10.18632/oncotarget.1905 (PMC4279378; doi:10.18632/oncotarget.1905)
Supplement: Supplementary file 1 [file oncotarget-05-10356-s001.pdf]

Poly(ADP-ribosyl)ation is involved in the epigenetic control of *TET1* gene transcription

SUPPLEMENTARY MATERIAL

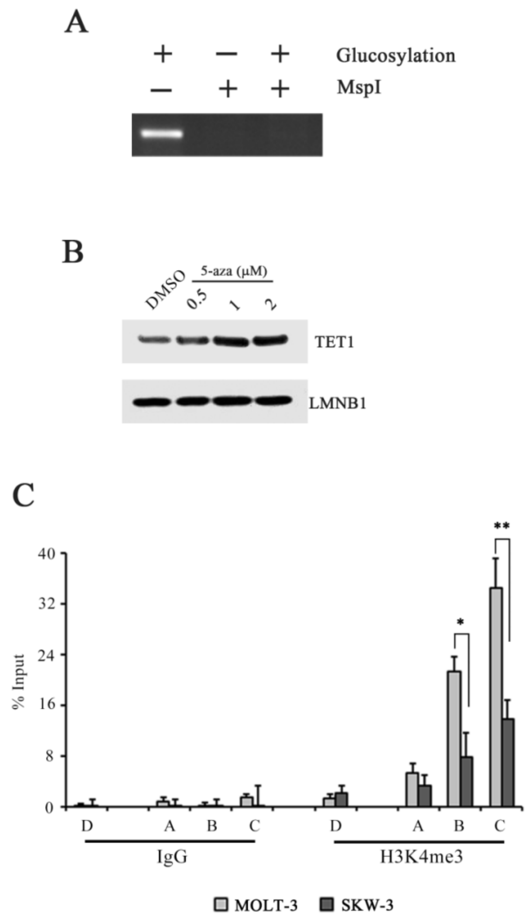

Figure S1: (**A**) Analysis of DNA hydroxymethylation of *TET1* CGI in SKW-3 cells performed using glucosyltransferase reaction and MspI restriction followed by PCR amplification with the same primers described in Figure 1. (**B**) Western blot analysis performed on total protein lysates showing TET1 expression after 5-azacytidine (5-aza) treatment of SKW-3 cells. LAMIN B1 (LMNB1) was used as loading control. (**C**) ChIP assay followed by qPCR performed on *TET1* regulative regions in MOLT-3 and SKW-3 cells. Localization of analysed fragments is shown in figure 3A. Antibodies against trimethyl histone H3K4 were used. The results are shown as percentage of input and are means ± S.E.M. (n=3). P-value was determined by Student's t-test (*P<0.05; **P<0.01)

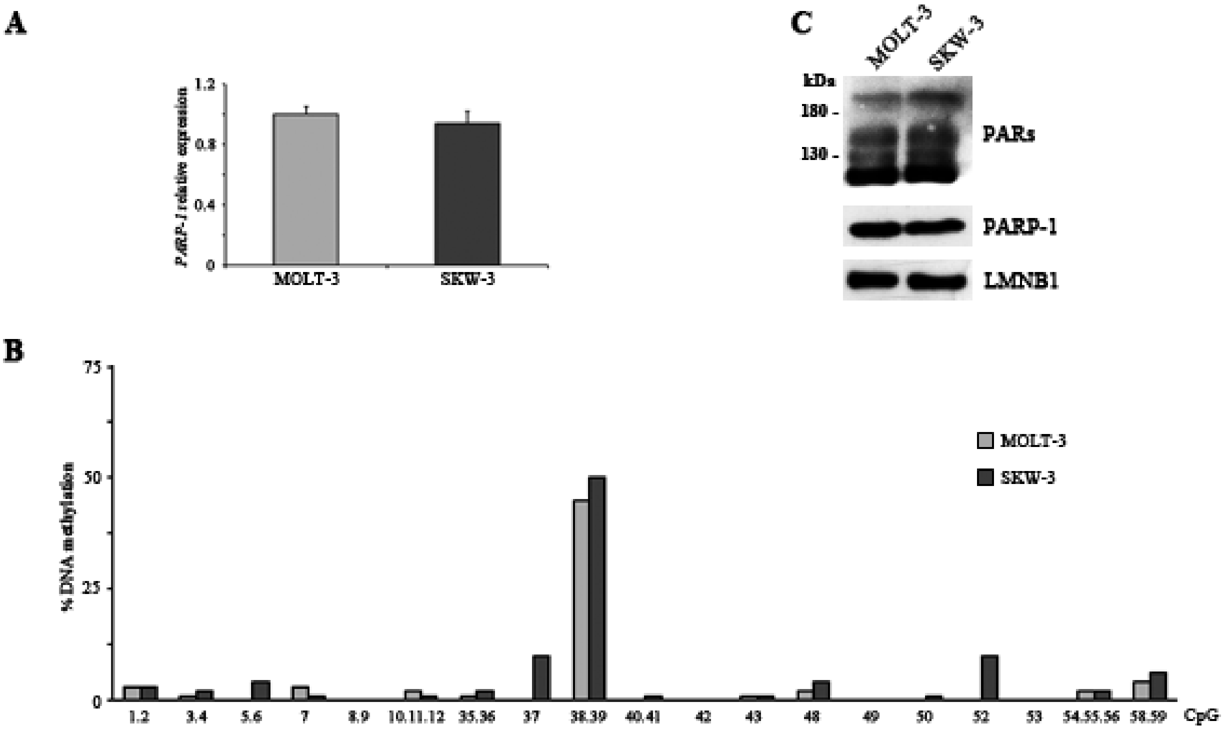

Figure S2: (**A**) qRT-PCR analysis of *PARP-1* gene expression. The results are shown as means ± S.E.M. (n=3). Statistical analysis was performed by using Student’s t-test but no statistical significance was obtained. (**B**) DNA methylation analysis of *PARP-1* CGI performed by using EpiTYPER assay. (**C**) Western blot analysis performed on total protein lysates showing PARP-1 and PARs. LAMIN B1 (LMNB1) was used as loading control.

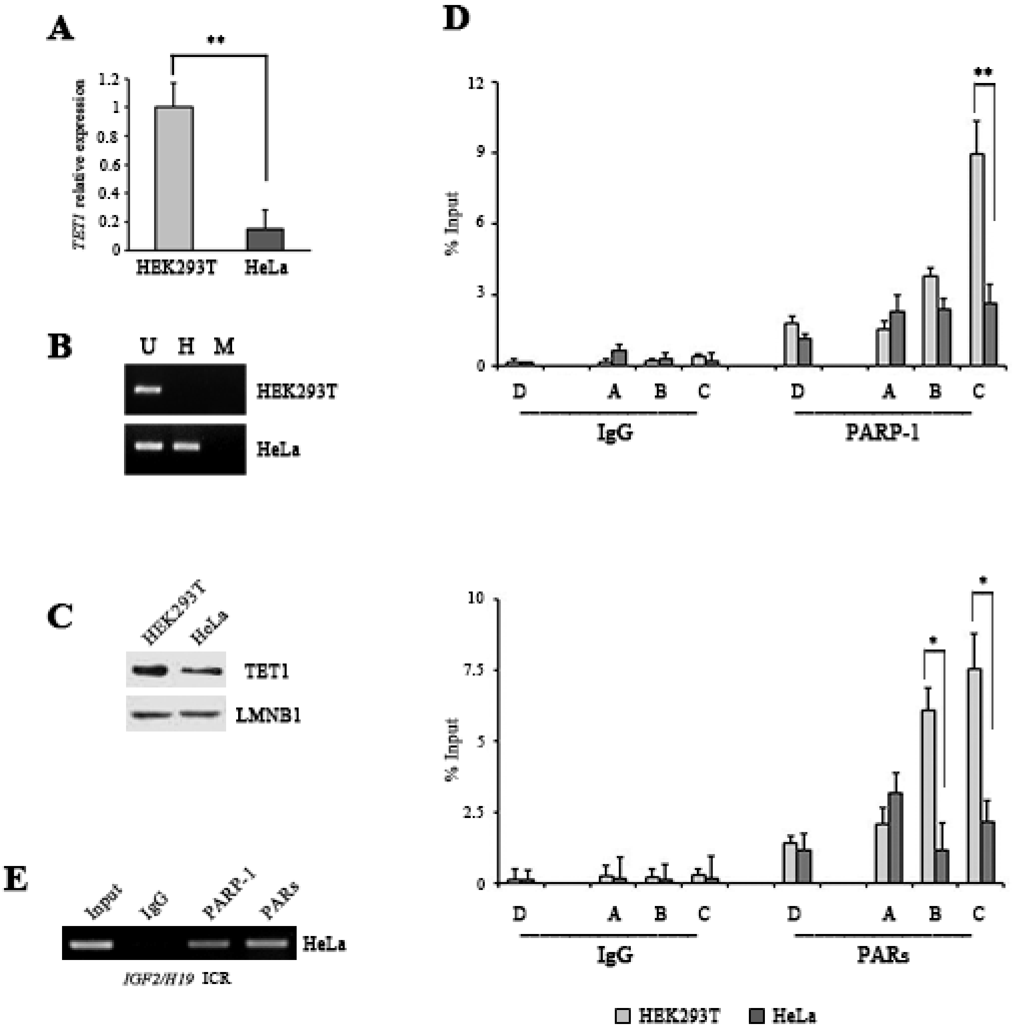

Figure S3: (**A**) qRT-PCR analysis of *TET1* gene expression in HEK293T and HeLa cells. The results are shown as means ± S.E.M. (n=3). P-value was determined by Student's t-test (**P<0.01). (**B**) Analysis of *TET1* DNA methylation using MspI/HpaII restriction and PCR amplification performed on HEK293T and HeLa cells. U, uncut; H, HpaII; M, MspI. (**C**) Western blot analysis performed on total protein lysates showing TET1 protein level in HEK293T and HeLa cells. LAMIN B1 (LMNB1) was used as loading control. (**D**) Analysis of PARP-1 and PARs presence on *TET1* gene regulative regions performed by ChIP assay followed by qPCR in HEK293T and HeLa cells. The results, shown as percentage of input, are means ± S.E.M. (n=3). P-value was determined by paired Student's t-test (*P<0.05; **P<0.01) (**E**) ChIP assay for PARP-1 and PARs on *Igf2/H19* imprinting control region (ICR) was used as positive control of immunoprecipitation in HeLa cells.

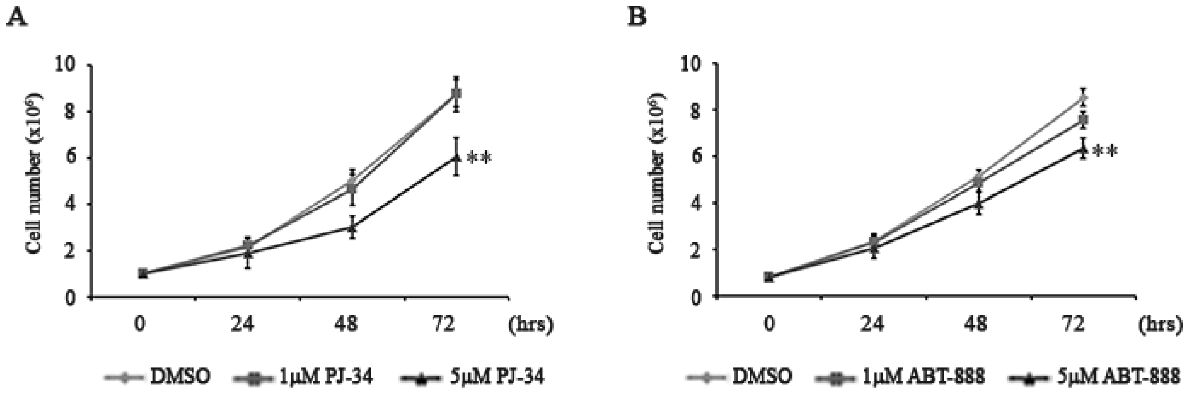

Figure S4: (**A-B**) Cell growth analysis of MOLT-3 cells treated with PJ-34 or ABT-888 determined by direct counting with trypan blue exclusion. The results are shown as percentage of input and are means ± S.E.M. (n=4). P-value was determined by paired Student's t-test (**P<0.01) comparing control (DMSO) cells with PARP-inhibited cells.

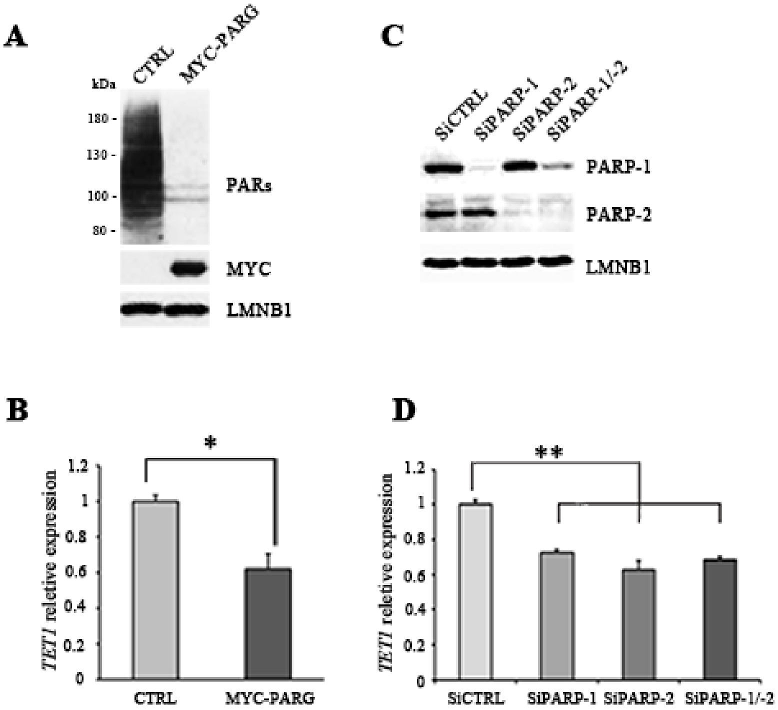

Figure S5: (**A**) Western blot analysis performed on total protein lysates of HEK293T cells overexpressing MYC-PARG or transfected with empty vector (CTRL). LAMIN B1 (LMNB1) was used as loading control. (**B**) qRT-PCR analysis of *TET1* gene expression in HEK293T transfected with MYC-PARG or empty vector (CTRL). The results are shown as means ± S.E.M. (n=3). (**C**) Western blot analysis performed on total protein lysates of HEK293T cells silenced for PARP-1 (siPARP-1), PARP-2 (siPARP-2) and both (siPARP-1 /-2), or transfected with non-targeting siRNA (CTRL). LMNB1 was used as loading control. (**D**) qRT-PCR analysis of *TET1* gene expression in HEK293T transfected with CTRL or PARP-1 and PARP-2 siRNA . The results are shown as means ± S.E.M. (n=3). P-value was determined by paired Student’s t-test (*P<0.05; **P<0.01).

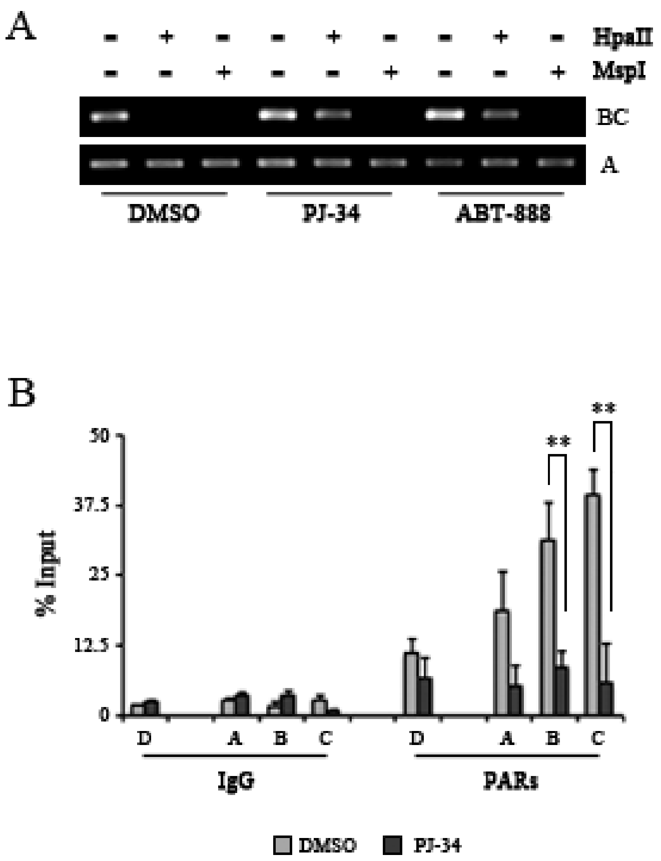

Figure S6: (**A**) Analysis of *TET1* DNA methylation using MspI/HpaII restriction and PCR amplification performed on MOLT-3 cells treated for 96 hrs with 1μM PJ-34 or 1μM ABT-888. Localization of analysed fragments is shown in figure 4A, where BC fragment is the region of interest while A fragment was used as loading control. (**B**) ChIP assay followed by qPCR performed on *TET1* regulative regions in MOLT-3 cells treated with 1μM PJ-34 for 72 hrs. Localization of analysed fragments is shown in figure 3A. Antibodies against PARs were used. The results, shown as percentage of input, are means ± S.E.M. (n=3). P-value was determined by paired Student's t-test (**P<0.01).

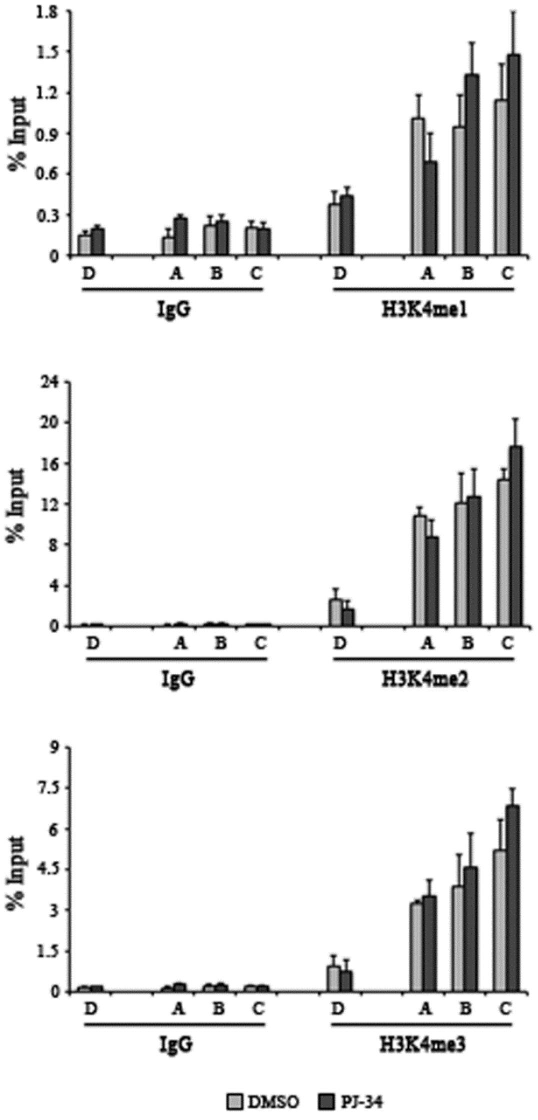

Figure S7: ChIP assay followed by qPCR performed on *TET1* regulative regions in SKW-3 cells treated with 1μM PJ-34 for 72 hrs. Localization of analysed fragments is shown in figure 3A. Antibodies against mono-, di- and trimethyl histone H3K4 were used. The results are shown as percentage of input and are means ± S.E.M. (n=3). Statistical analysis was performed by paired Student's t-test.

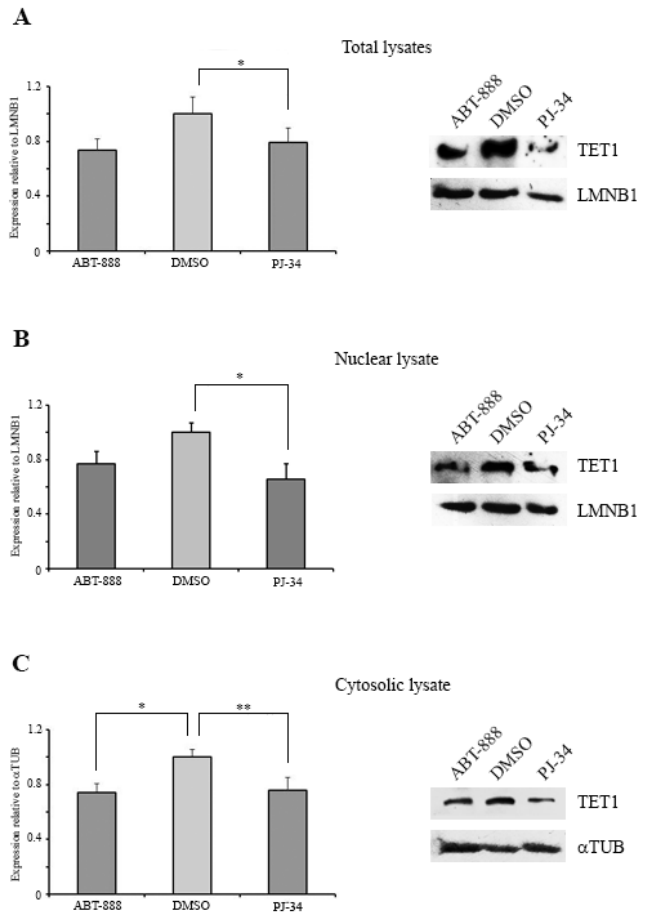

Figure S8: Densitometric analysis of TET1 protein levels in MOLT-3 after 72 hrs inhibition with 1μM PJ-34 and 1μM ABT-888 in total protein lysates (**A**), nuclear (**B**) and cytosolic (**C**) fractions. Four independent experiments were performed and representative western blots are shown. LAMIN B1 (LMNB1) and αTUBULIN (αTUB) were used as loading control and for normalization in densitometric analysis. The results are shown as means ± S.E.M. (n=4). P-value was determined by paired Student's t-test (*P<0.05; **P<0.01).

SUPPLEMENTARY METHODS

Primers used for ChIP assays

Primer	Sequence
h*TET1*-ChIP-A F	CTAGAGCTCCCCTGGGAAAA
h*TET1*-ChIP-A R	CCCTCGTTCTCCACCTCTCT
h*TET1*-ChIP-B F	CCAGCTCCAGTTTGGGTAAA
h*TET1*-ChIP-B R	GGACAGACCTCAGGGAGTGA
h*TET1*-ChIP-C F	CAAGTCATGCAGCCCTACCT
h*TET1*-ChIP-C R	GGCTCCCAGCACAGTCAA
h*TET1*-ChIP-D F	TCTGAATAGGCATGTATGGAGGT
h*TET1*-ChIP-D R	CCATTCATTTTCACCTGAGTGAT
h*H19ICR*-ChIP F	CCCATCTTGCTGACCTCAC
h*H19ICR*-ChIP R	AGACCTGGGACGTTTCTGTG
h*HOXA9*-ChIP-A F	CCACCCTGCCTTGTTTCAACATCA
h*HOXA9*-ChIP-A R	ACCAAGTTGTCAGTGAGCCTTCCA
h*HOXA9*-ChIP-B F	TTCATCCTCACCAGCAGTTCCAGT
h*HOXA9*-ChIP-B R	GGGCCATTTCGGAGTTCATTGTGT

Taqman Gene Expression Assays

Gene	Assay ID
TET1	Hs00286756_m1
PARP-1	Hs00242302_m1
GUSB	Hs99999908_m1
ACTB	4352668

Primers used for EpyTYPER analysis

Primer	Sequence
TET1-1 F	AGGAAGAGAGAGGTAGGTAGGGTTGTATGATTTGG
TET1-1 R	CAGTAATACGACTCACTATAGGGAGAAGGCTCCCAACTTCACTCCCTAAAATCT AT

TET1-2 F	AGGAAGAGAGGGTTTTTAGTTTTAAGTTTGTATTAGTTTT
TET1-2 R	CAGTAATACGACTCACTATAGGGAGAAGGCTATCATACAACCCTACCTACCTCTCC
PARP-1 F	AGGAAGAGAGTTTGGGATAGAATAATTAAAGGGG
PARP-1 R	CAGTAATACGACTCACTATAGGGAGAAGGCTCACCTACACCATAATAACCATCC

Primers used for bisulfite sequencing

Primer	Sequence
BISO h*TET1* CGI F	AGGGATTTTAGTTTTAGTTTGGGTAAA
BISO h*TET1* CGI R	CCAAAAATCCACAATAAAAAAACA

Primers used for PCR analysis after HpaII/MspI restriction

For this analysis amplification was obtained using primers listed in ChIP analysis table:

Fragment A: h*TET1*-ChIP-A F; h*TET1*-ChIP-A R
Fragment BC: h*TET1*-ChIP-B F; h*TET1*-ChIP-C R
